# Production of Hydrogen Sulfide from D-Cysteine and Its Therapeutic Potential

**DOI:** 10.3389/fendo.2013.00087

**Published:** 2013-07-16

**Authors:** Norihiro Shibuya, Hideo Kimura

**Affiliations:** ^1^Department of Molecular Pharmacology, National Institute of Neuroscience, Kodaira, Tokyo, Japan

**Keywords:** hydrogen sulfide, bound sulfane sulfur, l-cysteine, d-cysteine, 3MST, DAO, ischemia-reperfusion injury

## Abstract

Accumulating evidence shows that H_2_S has physiological functions in various tissues and organs. It includes regulation of neuronal activity, vascular tension, a release of insulin, and protection of the heart, kidney, and brain from ischemic insult. H_2_S is produced by enzymes from l-cysteine; cystathionine β-synthase, cystathionine γ-lyase, and 3-mercaptopyruvate sulfurtransferase (3MST) along with cysteine aminotransferase. We recently discovered an additional pathway for the production of H_2_S from d-cysteine. d-Amino acid oxidase provides 3-mercaptopyruvate for 3MST to produce H_2_S. d-Cysteine protects cerebellar neurons from oxidative stress and attenuates ischemia-reperfusion injury caused in the kidney more effectively than l-cysteine. This review focuses on a novel pathway for the production of H_2_S and its therapeutic application especially to the renal diseases.

## Introduction

The discovery of endogenous sulfide in the brain urged us to study the function of hydrogen sulfide (H_2_S) in the brain (1–3). The recent re-evaluation showed that the endogenous levels of H_2_S are much lower than those initially evaluated, but this finding confirmed the existence of sulfide in tissues (4–6).

**Figure 1:**
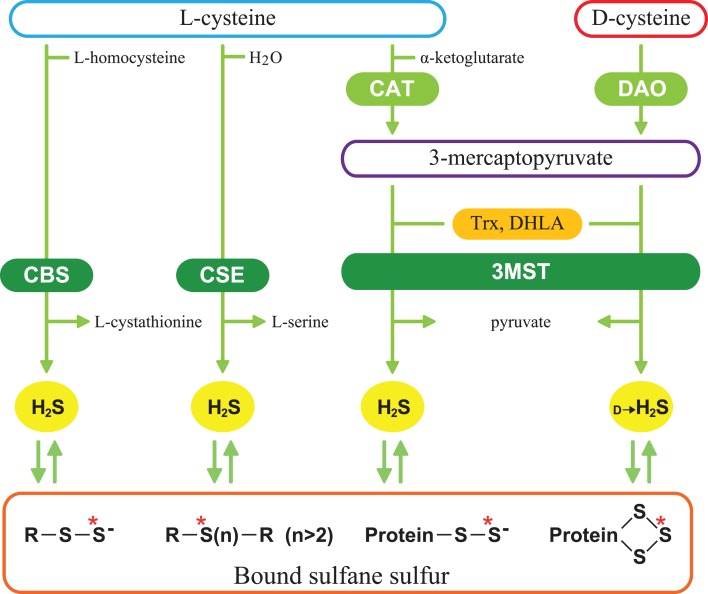
**Schematic representation of H_2_S-producing pathways**. Cystathionine β-synthase (CBS) catalyzes β-replacement of l-cysteine to produce H_2_S and l-cystathionine. Cystathionine γ-lyase (CSE) catalyzes the hydrolysis of l-cysteine. 3-Mercaptopyruvate sulfurtransferase (3MST) produces H_2_S from 3-mercaptopyruvate (3MP), which is generated by cysteine aminotransferase (CAT) and d-amino acid oxidase (DAO) from l-cysteine and d-cysteine, respectively. Thioredoxin (Trx) and dihydrolipoic acid (DHLA) are endogenous reducing cofactors that facilitate the release of H_2_S from 3MST. H_2_S is stored as bound sulfane sulfur, which is divalent sulfur bound only to other sulfur, such as outer sulfur atoms of persulfides and innerchain atoms of polysulfides. Red asterisks show bound sulfane sulfur.

H_2_S facilitates the induction of hippocampal long-term potentiation, a synaptic model of learning and memory, by enhancing the activity of *n*-methyl-d-aspartate (NMDA) receptors in neurons, and it induces Ca^2+^ waves in astrocytes (7, 8). It relaxes vascular smooth muscle by activating K^+^ channels, regulates the release of insulin and induces angiogenesis (9–14). It protects neurons from oxidative stress by enhancing the activity of glutathione synthesis, scavenging reactive oxygen species, and suppressing the excessive increase in the intracellular Ca^2+^ (15–17). In cardiovascular system, H_2_S protects cardiomyocytes from ischemia-reperfusion injury by preserving mitochondrial function (18). A similar protective effect was also observed in the kidney (19). H_2_S is produced from l-cysteine by two pyridoxal 5′-phosphate (PLP)-dependent enzymes, cystathionine β-synthase (CBS), and cystathionine γ-lyase (CSE) and PLP-independent 3-mercaptopyruvate sulfurtransferase (3MST) (Figure 1) (7, 9, 20–23). 3MST produces H_2_S from 3-mercaptopyruvate (3MP), an achiral α-keto acid, which is generated by PLP-dependent cysteine aminotransferase (CAT) from l-cysteine and α-ketoglutarate (α-KG) (24–26). Thioredoxin (Trx) and dihydrolipoic acid (DHLA) are endogenous reducing cofactors that facilitate H_2_S release from 3MST (23). We recently discovered a novel pathway with d-cysteine as a substrate (27).

## Production of H_2_S from d-Cysteine

When we examined the production of H_2_S from brain homogenates, we found that H_2_S was produced from d-cysteine, originally used as a negative control for l-cysteine (27). H_2_S-producing pathway from d-cysteine is distinct from the pathways involving l-cysteine. There are critical differences between the two pathways; (i) the optimal pH, (ii) the dependency on PLP, and (iii) the stability against the freeze and thaw procedure. The production of H_2_S from d-cysteine is optimal at pH 7.4, whereas production from l-cysteine is maximal under the alkaline condition. H_2_S production from d-cysteine is PLP-independent, while that from l-cysteine is PLP-dependent. A single freeze-thaw cycle greatly increases the H_2_S production from d-cysteine. d-Amino acid oxidase (DAO) that produces 3MP from d-cysteine is localized to peroxisomes, while 3MST is mainly found in mitochondria (21, 28). Mitochondria and peroxisomes exchange various metabolites via a specific form of vesicular trafficking, and are usually in close proximity to each other or have physical contact (29). 3MST and DAO can produce H_2_S by the interaction of both organelles.

## Localization of H_2_S-Producing Enzymes

Enzymes producing H_2_S from l-cysteine are expressed in many tissues (7, 9, 17, 20, 21, 23, 30, 31). 3MST is found in neurons in the cerebral cortex, cerebellum, olfactory bulb, pons, and retina, while CBS is preferentially expressed in cerebellar Bergmann glia and in astrocytes throughout the brain (21, 32). CSE activity in the brain is only 1% of the hepatic activity (33). CBS, CSE and 3MST, and CAT are expressed in the liver and kidney (20). Vascular endothelium co-expresses 3MST and CAT (31). The localization of CSE in vascular endothelium is controversial (31, 34). Unlike the l-cysteine pathways, the d-cysteine pathway operates predominantly in the cerebellum and the kidney (27, 35). In the cerebellum, DAO is expressed in astrocytes, Bergmann glia, and several types of neurons including the Golgi and Purkinje cells (35, 36). In the kidney, DAO and 3MST are expressed in the proximal convoluted tubules of the cortex similarly to CBS and CSE (30, 37–39).

## Regulation of H_2_S-Producing Enzymes by Ca^2+^

3MST/CAT is regulated by Ca^2+^; the activity is maximal in the absence of Ca^2+^ and is completely suppressed at 2.9 μM Ca^2+^ (17). A similar regulation by Ca^2+^ is observed in CSE activity (40). H_2_S is produced by CSE at the steady-state low Ca^2+^ concentrations and that the production is suppressed by increased Ca^2+^ (40). Calmodulin is not involved in the regulation of CSE activity. It was previously reported that CSE activity is regulated by Ca^2+^/calmodulin in the presence of 1–2 mM Ca^2+^ (34). Because the intracellular Ca^2+^ concentrations are between 100 nM and 3 μM in endothelium, Ca^2+^ concentrations used in the previous study are not in the physiological range (41).

## Source of d-Cysteine

Relatively large amounts of d-serine are found in mammalian tissues, and the content of d-serine is up to 15 ∼ 30% of the l-form in the brain (42, 43). d-Serine is thought to be produced by PLP-dependent serine racemase, but the Michaelis-constant value of serine racemase is higher than the endogenous levels of l-serine (42, 44–46). Although cysteine is structurally similar to serine with an OH replaced by an SH, serine racemase does not change l-cysteine to d-cysteine (27). Aspartate racemase is homologous to CAT and has an affinity for both aspartate and cysteine (24, 47), but does not produce d-cysteine.

A possible source of d-cysteine is absorption from food. l-Amino acids are non-enzymatically racemized by heat and alkaline treatment applied during food processing. l-Cysteine is one of the fastest racemizing amino acid, and 21–44% of l-cysteine is changed to d-cysteine by alkaline treatment (48, 49). Although d-cysteine is easily absorbed through the gastrointestinal tract and enters the blood stream (50), d-cysteine is not detected either in the cerebellum or the kidney after the oral administration. Considering the fact that the levels of bound sulfane sulfur, a storage form of H_2_S (Figure 1), are increased after oral administration of d-cysteine (5, 27), d-cysteine may be immediately metabolized to produce bound sulfane sulfur in tissues.

## Cytoprotective Effect of d-Cysteine

The most characteristic feature of the d-cysteine pathway is the greater H_2_S-producing activity in the cerebellum and the kidney compared to the l-cysteine pathway; 7- and 80-fold greater in the cerebellum and the kidney, respectively. Although both d-cysteine and l-cysteine protect cerebellar neurons from hydrogen peroxide-induced oxidative stress (27), d-cysteine protected neurons more greatly than l-cysteine, probably because the transport activity for d-cysteine is greater than that for l-cysteine (51). d-Cysteine may have a potential to improve the developmental neuronal diseases in the cerebellum like autism in which oxidative stress may be involved (52, 53).

Ischemia-reperfusion injury is observed after cardiovascular surgery, transplantation, or septic as well as hemorrhagic shock. Renal ischemia-reperfusion injury reduces the filtering capacity of the glomerulus and causes acute renal failure (54). Endothelin antagonists, atrial natriuretic peptides, prostaglandins, nitric oxide inhibitors, thyroxine, and human insulin-like growth factor 1 have been studied for the prophylaxis and treatment of acute tubular necrosis without clinical benefit (55–58). We found that the oral administration of d-cysteine attenuates renal ischemia-reperfusion injury (27). The structure of glomeruli, which is disintegrated after ischemia-reperfusion, is well preserved by d-cysteine. In contrast, the glomeruli are shrunk and a wide space is observed between glomerulus and the surrounding capsule after ischemia-reperfusion when l-cysteine is applied. d-Cysteine increases the levels of bound sulfane sulfur and protects the renal cortex from the ischemia-reperfusion injury more efficiently than l-cysteine.

## d-Cysteine: Its Therapeutic Potential

l-Cysteine is metabolized to produce (i) cysteinyl-tRNA by cysteinyl-tRNA synthetase (59), (ii) γ-glutamylcysteine, a precursor of glutathione, by γ-glutamylcysteine synthetase, (iii) taurine or pyruvate by cysteine dioxygenase (60), and (iv) propionyl CoA by α-keto acid dehydrogenase. Because d-cysteine is not metabolized by these enzymes, d-cysteine must efficiently be utilized to produce H_2_S in the cerebellum and the kidney.

l-Cysteine is an excitotoxin comparable in potency to other excitatory amino acids and increases the blood pressure and heart rate (61, 62). In contrast, d-cysteine neither causes excitotoxic damage to the brain nor disturbs heart function (63, 64). Therefore, d-cysteine can be systemically and repeatedly applied with less toxicity compared to l-cysteine. The administration of d-cysteine may provide a new therapeutic approach to protect specific tissues from oxidative stress or ischemia-reperfusion injury through its conversion to H_2_S via a novel pathway with 3MST and DAO.

## Conflict of Interest Statement

The authors declare that the research was conducted in the absence of any commercial or financial relationships that could be construed as a potential conflict of interest.
